# Correlation of Serum Electrolyte Imbalances With Diabetic Duration and Medication Use: A Cross-Sectional Comparative Study

**DOI:** 10.7759/cureus.70065

**Published:** 2024-09-24

**Authors:** Bhagwant G Pawar, Madhavi Eerike, Anand K Pyati, Sakthivadivel Varatharajan, Kalpana Mali, Venu Gopala R Konda

**Affiliations:** 1 Pharmacology, All India Institute of Medical Sciences, Bibinagar, IND; 2 Biochemistry, All India Institute of Medical Sciences, Bibinagar, IND; 3 Internal Medicine, All India Institute of Medical Sciences, Bibinagar, IND; 4 Pharmacology, Neelima Institute of Medical Sciences, Anurag University, Hyderabad, IND

**Keywords:** anti-diabetic drugs, serum chloride, serum electrolytes, serum sodium, type 2 diabetes

## Abstract

Background: Certain anti-diabetic medications may exacerbate electrolyte imbalances, potentially complicating glycemic control in diabetic patients. The present study aimed to correlate the serum electrolyte imbalances such as Na^+^, K^+^, Ca^+2^, Cl^-^, and Mg^+2^ with the duration of disease, glycemic control, and medication regimens.

Method: In this cross-sectional study, 31 patients with type 2 diabetes mellitus (T2DM) and 30 healthy controls, with mean ages of 52.06 and 48.5 years, respectively, were recruited based on eligibility criteria. Data on demographic information, medication history, and duration of diabetes were collected. Fasting blood sugar (FBS), postprandial blood sugar (PPBS), glycated hemoglobin (HbA1C), and serum electrolytes were measured. The data were statistically analyzed. The mean differences in serum electrolytes between T2DM patients and non-diabetic participants were compared using the Mann-Whitney U test, and correlation analysis was performed. A p-value of <0.05 was considered statistically significant.

Result: Around 9.6% of participants had diabetes duration of less than one year, while the majority (45%) fell within the 1-5-year duration range. Most diabetic patients (61.2%) exhibited poor glycemic control. Statistically significant differences were observed between the mean FBS, PPBS, and HbA1C levels of T2DM (150, 249, and 8.82, respectively) and control group (95, 114, and 5.52, respectively). Analysis of serum electrolytes showed statistically significant differences with regard to Na^+^, K^+^, and Cl^-^ between the diabetic and control groups. Mean sodium and chloride levels were lower and potassium levels were higher in diabetic patients compared to the control group. Negative correlations were observed between sodium and chloride levels and duration of diabetes and HbA1C levels.

Conclusion: The study reveals significant electrolyte imbalances in patients with T2DM, characterized by reduced sodium and chloride levels and elevated potassium levels compared to healthy controls. These alterations are closely associated with poor glycemic control and longer disease duration, emphasizing the importance of regular electrolyte monitoring in T2DM management to mitigate potential complications.

## Introduction

Diabetes mellitus (DM), characterized by chronic hyperglycemia, is a prevalent metabolic disorder, with notable prominence in India, often referred to as the diabetes capital of the world. With 74.1 million cases in India alone in 2021 [1] and 422 million worldwide [2], the management of diabetes remains a critical healthcare challenge. Type 2 diabetes mellitus (T2DM) is diagnosed when the HbA1c level is ≥6.5%, fasting blood sugar (FBS) is ≥126 mg/dL, or random blood sugar (RBS) is ≥200 mg/dL, according to the American Diabetes Association (ADA) criteria [3].

In the realm of diabetes management, a multitude of anti-diabetic drugs, both oral and parenteral, are employed to regulate blood glucose levels. However, the intricate interplay between these medications and electrolyte balance warrants careful consideration. Elevated blood glucose levels drive plasma osmolarity, triggering the movement of water from intracellular to extracellular spaces [4], thus diluting electrolyte concentrations. Moreover, certain anti-diabetic medications may further exacerbate electrolyte imbalances, potentially complicating glycemic control in diabetic patients. Understanding these dynamics is crucial for optimizing treatment strategies and mitigating potential complications.

Research by Mustafa et al. in Sudanese populations revealed elevated serum potassium and chloride levels in type 2 diabetes (T2D) patients compared to healthy individuals [5], underscoring the pertinence of electrolyte management in diabetes care. Furthermore, T2D itself predisposes individuals to electrolyte imbalances, particularly potassium derangement, which may impact renal function and contribute to hypertension (HTN), a known risk factor for electrolyte disorders [6].

A retrospective longitudinal study based on data from the National Registry of Chronic Kidney Disease in Colombia highlighted the heightened risk of poor glycemic control (PGC) in individuals with both DM and chronic kidney disease (CKD). Notably, the odds (OR: 1.78, CI 95%: 1.55-2.05) of PGC was 78% higher among those with CKD compared to those without, with obesity further amplifying the risk by 52% [7].

Abnormal serum electrolyte levels in diabetic patients may contribute to PGC and increase the risk of diabetic complications. Managing these conditions often requires the use of multiple anti-diabetic drugs (more than two). Several studies have explored serum electrolyte imbalances in patients with T2D, highlighting the complex interplay between electrolyte disturbances and diabetic complications. Hypomagnesemia has been widely reported among T2D patients, with evidence suggesting that low magnesium levels are associated with PGC and an increased risk of cardiovascular complications [8]. Serum sodium and potassium level abnormality was observed in most of the studies [9-11]. Despite the growing body of research on electrolyte disturbances in T2D, few studies have directly correlated these imbalances with specific anti-diabetic medications. Most research to date has focused on traditional drugs like insulin and sulfonylureas, while data on newer drug classes, such as SGLT2 inhibitors and GLP-1 receptor agonists, remains limited. Hence, the present study aims to assess serum electrolytes in diabetic patients receiving various anti-diabetic treatments and explore potential correlations with the duration of drug use.

## Materials and methods

A cross-sectional study design was employed to compare serum electrolyte levels between T2DM patients and age- and gender-matched healthy controls.

The study commenced after obtaining approval from the All India Institute of Medical Sciences Bibinagar Institutional Ethics Committee (AIIMS BBN-IEC) (approval number: AIIMS/BBNIEC/MAR/2023/264) and followed the principles of the Declaration of Helsinki. The study participants were screened from the general medicine department. They were recruited as per the inclusion and exclusion criteria. Participants aged 30-70 years, diagnosed with T2DM, currently receiving treatment with anti-diabetic medications (including both oral medications and injectables), and willing to participate in the study were included. Additionally, a control group of non-diabetic individuals matched for age was recruited to facilitate comparison. Acutely ill and hospitalized participants, participants using medications known to cause hyperglycemia, those diagnosed with type 1 diabetes mellitus (T1DM), participants using traditional medicine, participants with malignancies or infectious diseases, participants diagnosed with kidney disease, HTN, or asthma, and participants using diuretic medications were excluded from the study.

Written informed consent was obtained, and demographic details, duration of diabetes, and medication details (name, dose, frequency, and route of administration) were collected. The duration of diabetes was divided into <1 year, 1 to <5 years, 5 to <10 years, and >10 years. The diabetic control based on HbA1C was categorized into the following: <7: good glycemic control, 7-8: inadequate glycemic control, and >8: poor glycemic control. The control group was divided into normal and prediabetic based on HbA1C levels [3].

**Figure 1:**
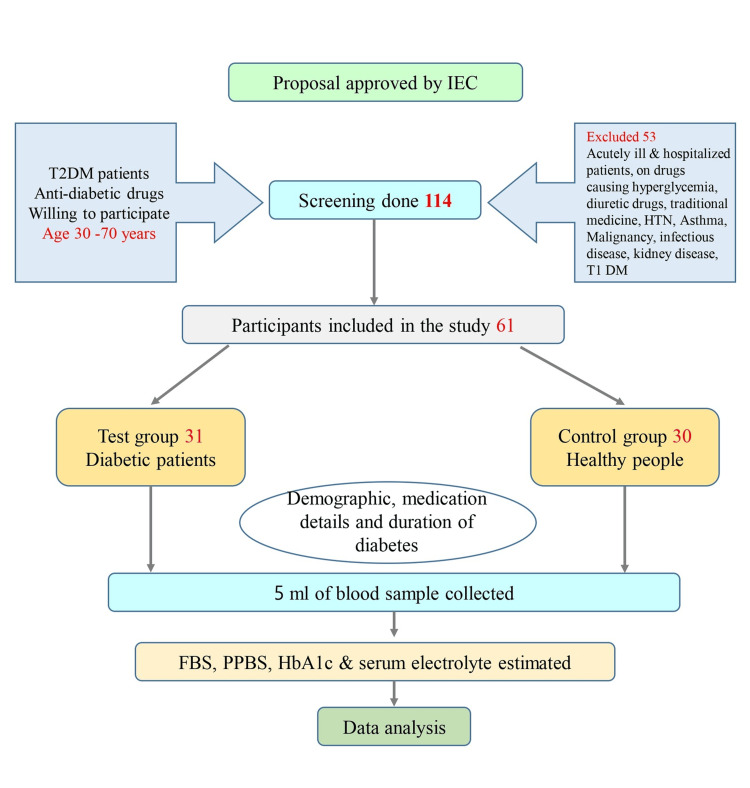
Flowchart of the study

Later from each participant, 3 ml of the venous blood sample was collected in a plain container and 2 ml in a fluoride container under aseptic precaution. Lab investigations were carried out after the centrifugation and separation of serum. FBS and postprandial blood sugar (PPBS) were measured by the glucose oxidase-peroxidase method, while HbA1C was measured by the high-performance liquid chromatography (HPLC) method. Serum electrolytes such as sodium (Na^+^), potassium (K^+^), chloride (Cl^-^), calcium (Ca^2+^), and magnesium (Mg^2+^) were measured using standard laboratory methods. Electrolyte imbalance is considered when there is a deviation from the normal limits: Na^+^ (135-145 mmol/L), K^+^ (3.5-5 mmol/L), Ca^2+^ (8.6-10.2 mg/dL), Cl^-^ (96-106 mmol/L), and Mg^2+^ (1.6-2.6 mg/dL). The detailed methodology is shown in Figure 1.

Sample size estimation

Based on an anticipated mean difference in sodium levels of 2.1 mEq/L with a standard deviation of 0.43 mEq/L, the study initially required a total of 106 participants (53 in each group) to achieve 80% power with a 5% significance level [12]. However, due to constraints in time and resources, it was challenging to recruit and manage the initially calculated sample size. As a result, the study was conducted with 60 participants. Given this limitation, we recalculated the sample size using the Alpha Beta software to reflect a reduced power of 70%. The recalculated sample size indicated that 78 participants (39 in each group) would be required to maintain adequate power.

Statistical analysis

Data were collected using a case report form. Descriptive statistics were used to summarize demographic details, duration of diabetes, classification of participants based on HbA1c levels, electrolyte abnormalities, and medication details. These were expressed as percentages. Serum electrolyte levels were presented as mean±SEM. The normality of the data was assessed using the Shapiro-Wilk test. For normally distributed data, mean differences in serum electrolytes between T2D and non-diabetic participants were compared using the Mann-Whitney U test as the data was not normally distributed. For correlation analysis, Spearman's rank correlation coefficient was applied for non-normally distributed data. A p-value of <0.05 was considered statistically significant.

## Results

The present study comprised 31 participants in the test group and 30 participants in the control group, with mean ages of 52.06 and 48.5 years, respectively. Participants in the control group were age-matched to those in the test group, resulting in no statistically significant difference in age distribution between the groups. A nearly equal number of males and females were included in the test group, whereas more females (n=23, 76.6%) were recruited in the control group. Body mass index (BMI) did not differ significantly between the groups, although the control group exhibited a slightly higher mean BMI (26 compared to 25.8 in the test group) (Table 1).

**Table 1:** Baseline characteristics of participants Values are expressed as percentages or mean±SD NA: not applicable; FBS: fasting blood sugar; PPBS: postprandial blood sugar

Characteristics	Test group				Control group				P-value
N	31				30				-
Age in years (mean±SD)	52.06±11.89				48.5±11.98				>0.05
Male n (%)	16 (51.6%)				7 (23.3%)				-
Female n (%)	15 (48.3%)				23 (76.6%)				-
BMI kg/m^2^ (mean±SD)	25.89±4.28				26.03±3.47				>0.05
Duration of diabetes									
<1 year	3 (9.6%)				NA				NA
1 to <5 years	14 (45.1%)				NA				NA
5 to <10 years	8 (25.8%)				NA				NA
>10 years	6 (19.3%)				NA				NA
HbA1C in %									
<5.7	0				17 (56.6%)				NA
5.7-6.5 (prediabetes)	0				13 (43.3%)				NA
<7 (good glycemic control)	5 (16.1%)				NA				NA
7-8 (inadequate glycemic control)	7 (22.5%)				NA				NA
>8 (poor glycemic control)	19 (61.2%)				NA				NA
FBS mg/dL (mean±SD) (95% CI)	150.64±63.59	95% CI: 127.32-173.97			95.1±14.82	95% CI: 89.565-100.63			<0.0001
PPBS mg/dL (mean±SD) (95% CI)	249.81±88		95% CI: 217.53-282.08		114.13±30.64		95% CI: 102.69-125.57		<0.0001
HbA1C % (mean±SD) (95% CI)	8.82±2.52			95% CI: 7.895-9.750	5.52±0.53			95% CI: 5.322-5.724	<0.0001

Regarding the duration of diabetes, only three participants (9.6%) had a duration of less than one year, while the majority (n=14, 45%) fell within the 1-5-year duration range. Most diabetic patients (n=19, 61.2%) exhibited poor glycemic control, with only five participants (16%) achieving an HbA1C level of less than 7%. In the control group, nearly 13 participants (43%) were in the prediabetic stage (Table 1).

Statistically significant differences were observed between the mean FBS, PPBS, and HbA1C levels of the test (150, 249, and 8.82, respectively) and control groups (95, 114, and 5.52, respectively). These differences indicate that the test group had higher blood sugar levels and poorer glycemic control compared to the control group (Table 1).

Analysis of serum electrolytes was done using the Mann-Whitney U test for Na^+^, Cl^-^, Ca^+2^, and Mg^+2^ and the independent t-test for K^+^, based on the normality of distribution. Results showed no statistically significant differences between the diabetic and control groups for Ca^+2^ and Mg^+2^ and significant differences with Na^+^, K^+^, and Cl^-^. However, mean sodium levels were slightly lower in diabetic patients (135.53) compared to the control group (139.55), while mean potassium levels were higher in diabetic patients (4.29) compared to the control group (3.96). Similarly, mean chloride levels were lower in diabetic patients (98.28) compared to the control group (102.48). Details are shown in Table 2.

**Table 2:** Serum electrolyte status between the test group and control group Values for Na^+^, K^+^, Cl^-^, Ca^+2^, and Mg^+2 ^are expressed as mean± SD with a 95% confidence interval, and electrolyte abnormalities are expressed as percentages *p<0.05 considered as statistically significant

Characteristics	Test group	Control group	P-value
Electrolyte name	31	30	NA
*Na^+^ mEq/L	135.53±7.61 (95% CI: 132.74-138.33)	139.55±3.34 (95% CI: 138.30-140.80)	0.0118
K^+^ mEq/L	4.29±0.42 (95% CI: 4.13-4.44)	3.96±0.43 (95% CI: 3.79-4.12)	0.0037
Cl^-^ mEq/L	98.28±4.91 (95% CI: -96.47-100)	102.48±3.17 (95% CI: -101.3-103.67)	<0.0001
Ca^+2^ mg/dL	9.09±0.49 (95% CI: 8.913-9.274)	9.39±0.63 (95% CI: 9.158-9.629)	0.0569
Mg^+2^ mg/dL	1.87±0.15 (95% CI: -1.81-1.92)	1.95±0.16 (95% CI: -1.89-2.0)	0.0638
At least one of the five electrolyte abnormalities n (%)	19 (61.2%)	8 (26%)	
Hypernatremia n (%)	1 (3.2)	1 (3.3)	NA
Hyponatremia n (%)	11 (35.5)	1 (3.3)	NA
Hyperkalemia n (%)	1 (3.2)	0 (0)	NA
Hypokalemia n (%)	0 (0)	0 (0)	NA
Hyperchloremia n (%)	1 (3.2)	0 (0)	NA
Hypochloremia n (%)	9 (29)	2 (6.6)	NA
Hypercalcemia n (%)	0 (0)	3 (9.9)	NA
Hypocalcemia n (%)	6 (19.3)	3 (9.9)	NA
Hypomagnesemia n (%)	1 (3.2)	0 (0)	NA

Percentage calculations revealed that the overall prevalence of electrolyte imbalance was 61.2% (n=19) in the diabetic group and 26% (n=8) in the control population. Higher incidences of hyponatremia (n=11, 35.5%), hypochloremia (n=9, 29%), and hypocalcemia (n=6, 19.3%) were observed in the diabetic group compared to the control group. In the control group, hyponatremia (n=1, 3.3%), hypochloremia (n=2, 6.6%), and hypo- and hypercalcemia (n=3, 9.9% each) were observed.

Correlation analyses in the diabetic group revealed statistically significant positive correlations between FBS and HbA1C (r=0.682) and PPBS and HbA1C (r=0.609), indicating a moderately strong positive linear relationship between blood sugar levels and HbA1C.

Regarding medication usage, the majority of participants (n=8, 25.8%) were on a combination of metformin and glimepiride, followed by metformin alone (n=7, 22%). Only two participants (3.2%+3.2%) were on a four-drug combination regimen. Duration of use was expressed in years and values were expressed as ranges from minimum to maximum duration of use (Table 3).

**Table 3:** Percentage of drugs or drug combinations used by the test group + indicates fixed combination; "and" indicates individual drugs. The duration was given as a range

Drugs	Percentage use	Dose in mg	Frequency	Duration of use in years
Metformin n (%)	7 (22.5%)	500	OD(5)/ BD(2)	0.5-12
Metformin+gliclazide n (%)	2 (6.4%)	500+80	OD/BD	2-3
Metformin+glipizide n (%)	1 (3.2%)	250+2.5	OD	1
Metformin+glimepiride n (%)	8 (25.8%)	500+2.5	OD/BD	0.25-13
Metformin+sitagliptin n (%)	2 (6.4%)	1000+50 500+50	OD BD	0.25-0.5
Metformin+teneligliptin n (%)	2 (6.4%)	500+20	OD	1-2
Metformin and insulin n (%)	1 (3.2%)	500 and 4 IU	OD	Metformin: 8; insulin: 1
Gliclazide and dapagliflozin n (%)	1 (3.2%)	60 and 10	OD	2
Sitagliptin+glimepiride+metformin n (%)	1 (3.2%)	50+2+1000	OD	3
Metformin+glimepiride+voglibose n (%)	4 (12.9%)	500+2+0.2	OD	0.5-4
Metformin+vildagliptin and dapagliflozin and glimepiride n (%)	1 (3.2%)	500+50 and 10 and 4	OD	1
Metformin+glimepiride+voglibose and dapagliflozin n (%)	1 (3.2%)	500+2+0.2 and 10	OD	2

Insulin and metformin usage was associated with hyponatremia, hypochloremia, hypocalcemia, and hypomagnesemia. Additionally, hypernatremia (159.63) and hyperchloremia (114.4) were observed in patients on a combination of voglibose, glimepiride, and metformin for four years. The duration of medication usage also influenced electrolyte levels, with short-term use resulting in decreased levels and long-term use leading to hyperchloremia.

**Figure 2:**
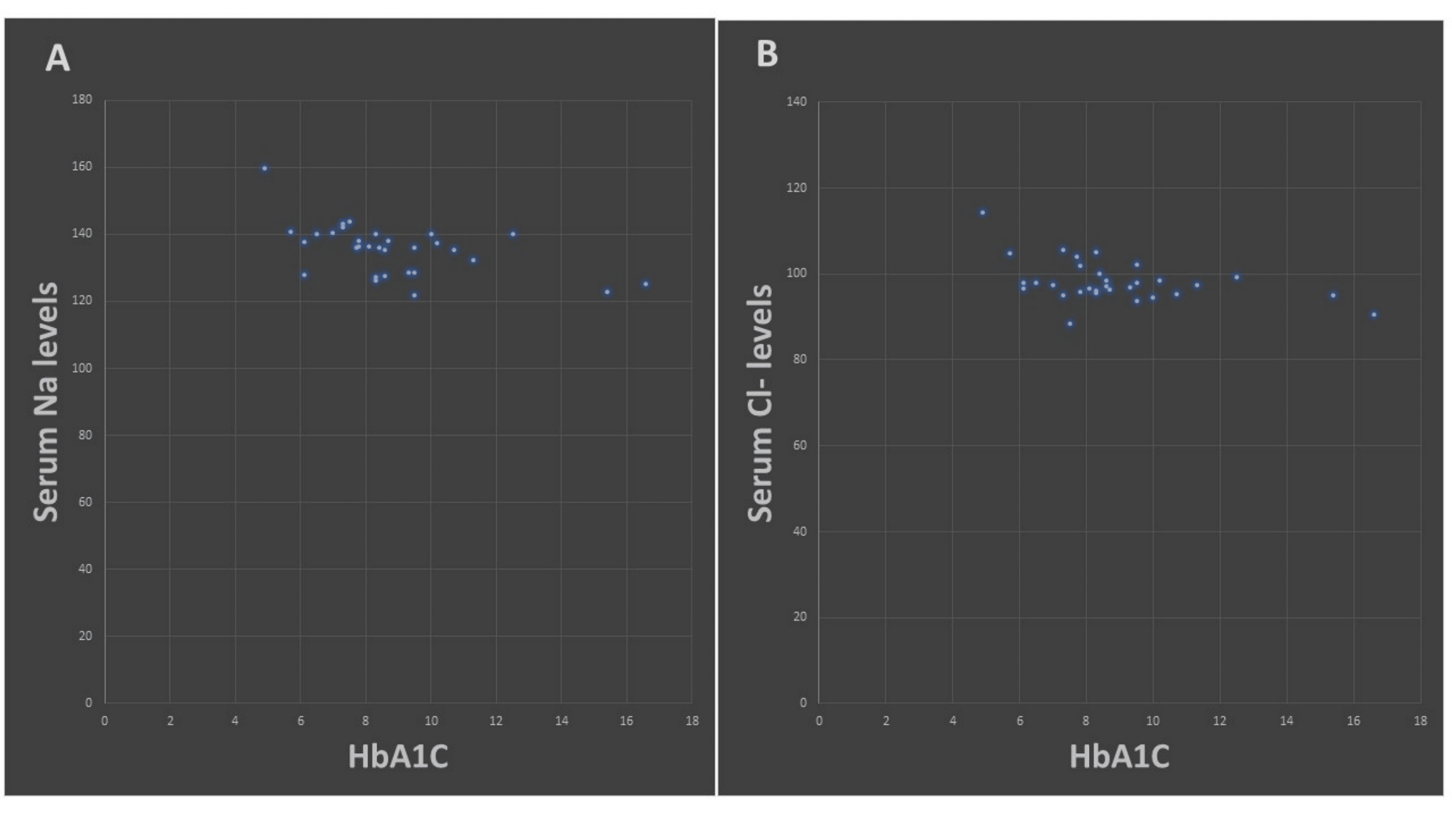
Scatter plot showing (A) the correlation of Na+ levels with HbA1C and (B) the correlation of Cl- levels with HbA1C

Correlation analyses between serum electrolytes, medication usage, duration of diabetes, and HbA1C levels revealed statistically significant negative correlations between HbA1C and sodium and chloride levels. The duration of diabetes also showed negative correlations with sodium and chloride levels, although these were not statistically significant. Correlation for Mg^+2^ was not done as it was observed in only one patient (Table 4 and Figure 2).

**Table 4:** Correlation of serum electrolytes (Na+, K+, Ca+2, Cl-) with drug/drug combinations, duration, and HbA1C levels in diabetic patients *p<0.05 considered as statistically significant

Character	Na^+^		K^+^		Ca^+2^		Cl^-^	
	r-value	P-value	r-value	P-value	r-value	P-value	r-value	P-value
Drug/drug combination	0.1254	0.5016	0.1760	0.3436	0.4067	0.0232*	0.01565	0.9334
Duration of diabetes	-0.2502	0.1747	0.08816	0.6372	0.0638	0.7329	-0.01156	0.9508
HbA1C	-0.5047	0.0038*	0.03069	0.8698	-0.2307	0.2117	-0.3622	0.0452*

## Discussion

The current study aimed to investigate the potential influence of anti-diabetic drugs on serum electrolyte status. While there are numerous studies available on the relationship between serum electrolyte imbalances and diabetes, this study specifically focuses on the combined effect of diabetic duration, medication use, and glycemic control in a comprehensive manner. We enrolled 31 participants in the test group and 30 in the control, with similar age distributions. The majority of participants had diabetes for 1-5 years, with PGC being prevalent. Significant differences in FBS, PPBS, and HbA1C levels between groups indicated poorer glycemic control in the test group. Positive correlations were found between blood sugar levels and HbA1C. Metformin and glimepiride combination was the most common medication. Serum electrolyte analysis showed lower sodium, chloride, and calcium levels in diabetic patients. Diabetic patients exhibited higher incidences of electrolyte imbalances compared to controls. Insulin and metformin usage were associated with electrolyte imbalances, with long-term use leading to hyperchloremia. Correlation analyses revealed negative correlations between HbA1C and sodium/chloride levels.

In our study, we recruited an age-matched but not a gender-matched control group. Surprisingly, 43% of the control group were in the prediabetic stage based on HbA1C levels. This prevalence was higher compared to the Indian Council of Medical Research-India Diabetes (ICMR-INDIAB) [13] study in 2017 which reported a prevalence of 10.3% and a global prevalence of 6.2% in 2021 [14]. The high prevalence of prediabetes is an interesting observation from our study. We have included patient attenders who qualify for inclusion and exclusion criteria as the control group. Most of them are working as security and homemakers. Their working condition and female gender may be the reason for increased HbA1C levels. Additionally, 30% of the control population had FBS >100 (100-125 mg/dL) and were in the prediabetic stage, while 13.3% had PPBS in the prediabetic stage (140-199 mg/dL). Due to the limited sample size, we were unable to recruit sex-matched controls in our study. Consequently, we were unable to evaluate the potential influence of hormonal fluctuations on electrolyte balance and glycemic control.

In the test group, diabetic patients exhibited poorer glycemic control despite drug treatment, with significantly higher FBS, PPBS, and HbA1C levels. The mean HbA1c was 8.82±2.52, and nearly 61% had PGC, consistent with the results (73%) of a cross-sectional study conducted by Abera et al. [15]. Nearly 19% had HbA1C above 10. Glycemic control was defined according to HbA1c levels by the ADA, with levels <7% considered as "good" control, 7-8% as "inadequate" control, and >8% as "poor" control. The majority (55%) of the participants had diabetes for less than five years. 

Our study highlighted that 77% of diabetic patients were on a fixed-dose combination of anti-diabetic drugs, predominantly metformin and glimepiride. According to diabetes management guidelines, first-line therapy with metformin monotherapy is recommended, and if HbA1C target levels are not achieved, dual therapy with metformin plus sulfonylureas or DPP-4 inhibitors will be used [16]. Among DPP-4 inhibitors, sitagliptin and teneligliptin were commonly used. Teneligliptin, reported as a cost-effective second-line agent, is approved in India but not by the US FDA [17].

Our analysis of serum electrolytes revealed notable differences though statistically not significant between diabetic patients and controls, with diabetic patients exhibiting lower serum sodium, chloride, and calcium levels. The higher incidences of electrolyte imbalances, including hyponatremia, hypochloremia, and hypocalcemia, among diabetic patients underscored the need for the routine monitoring of electrolyte levels in this population.

The findings from a cross-sectional study have consistently shown a statistically significant decrease in serum sodium and chloride levels in uncontrolled DM, without alterations in potassium and magnesium levels [18]. Electrolyte imbalances are common in individuals diagnosed with T2DM and are typically multifaceted, often stemming from insulin deficiency observed in diabetic ketoacidosis and elevated blood sugar levels [19]. Research has also indicated a correlation between decreasing serum sodium and chloride levels and rising fasting blood glucose levels. The physiological basis for hyponatremia is well understood: as blood glucose levels increase, water moves out of cells, leading to decreased sodium levels [20].

Our study suggests an association between medication usage, particularly insulin and metformin, and electrolyte imbalances, underscoring the importance of considering medication regimens when assessing and managing electrolyte disturbances in diabetic patients. Additionally, our findings reveal correlations between glycemic control, serum electrolytes, and medication usage, indicating complex interrelationships that warrant further investigation. Although the duration of diabetes showed trends towards negative correlations with sodium and chloride levels, these were not statistically significant.

In a study by Khan et al., no significant association was found between age, gender, comorbidities, and drug history, but a significant inverse effect on fasting blood glucose with serum sodium was noted in the presence of age, gender, and BMI in an adjusted model [18]. Similarly, a cross-sectional study conducted in Ethiopia demonstrated significantly decreased mean sodium and median magnesium and calcium levels in diabetic patients, with an inverse relationship observed for chloride levels. Factors such as alcohol consumption, lack of formal education, and hyperglycemia showed significant associations with electrolyte imbalance.

In our study, hypochloremia was observed in 19.3% of cases. While the prevalence of electrolyte imbalance in our study was lower compared to other reports (83.07% and 52.31% among diabetic patients and controls, respectively), it was still notable, with 61.2% of diabetic patients and 26% of controls exhibiting electrolyte abnormalities [21]. Hyponatremia and hypochloremia were the most prevalent electrolyte abnormalities observed. These findings indicate the importance of monitoring electrolyte levels in diabetic patients, particularly those with PGC. Clinicians should consider interventions to address electrolyte imbalances alongside efforts to improve blood sugar management. The precise mechanisms underlying the negative correlation between HbA1c levels and sodium/chloride levels may vary and could involve factors such as changes in renal function due to hyperglycemia, altered hormone levels, or disturbances in fluid and electrolyte homeostasis.

Hyponatremia and hypochloremia were the major percentage of electrolyte abnormalities observed in our study. In our study, hyponatremia was 35.5%, whereas it was 40.6%, 33%, 45.3%, 63.3%, and 75% in studies conducted in Ethiopia, India, Benin, Bangladesh, and Kerala, respectively [22-26].

The studies conducted were in admitted diabetic patients or just observational studies without control or retrospective studies. Only one study was done in diabetic patients with a comparative group. Our study has correlated the electrolyte imbalance with diabetic control, duration of diabetes, and medication use. We have included only diabetic patients without any comorbidities. 

No study has correlated diabetic control status with electrolyte imbalance. A negative correlation was observed with sodium, calcium, and chloride which suggests an association between electrolyte imbalances and glycemic control. An increase in HbA1c levels, i.e., worsening glycemic control in diabetic patients, may cause alterations in electrolytes. It suggests that monitoring electrolyte levels may be important in diabetic patients, particularly those with PGC. Clinicians may need to consider interventions to address electrolyte imbalances alongside efforts to improve blood sugar management.

Limitations

Firstly, a sample size with a statistical power reduced to 70% increases the risk of type II errors (false negatives), where real differences between groups may not reach statistical significance. This could explain why, although trends in electrolyte imbalances (e.g., lower sodium and chloride and higher potassium levels) were observed, they did not achieve statistical significance. Additionally, the small sample size limits the generalizability of our results. As the study was conducted at a single institution with a specific patient population, the findings may not fully reflect the broader diabetic population. Lastly, gender differences are known to influence both electrolyte balance and glycemic control, potentially confounding the results. The absence of gender matching introduces the possibility of gender-related bias in our findings.

## Conclusions

This study highlights significant alterations in serum electrolyte levels among patients with T2DM compared to healthy controls. The findings indicate that diabetic patients tend to have PGC, as reflected by higher mean levels of FBS, PPBS, and HbA1C compared to the control group. Metformin and glimepiride were the most commonly prescribed medications for diabetes management. The analysis of serum electrolytes revealed statistical differences in serum sodium, potassium, and chloride levels between the two groups underscoring the potential impact of T2DM on electrolyte balance. Specifically, lower sodium and chloride levels and higher potassium levels were noted in diabetic patients, which could have clinical implications for the management of T2DM.

The negative correlations between sodium and chloride levels with both the duration of diabetes and HbA1C levels suggest that prolonged hyperglycemia may exacerbate electrolyte imbalances. These findings underscore the importance of regular monitoring of serum electrolytes in T2DM patients, particularly those with PGC, to prevent and manage potential complications. Further research with larger sample sizes is warranted to confirm these associations and explore the underlying mechanisms.
